# Gene Polymorphism of Aspirin-Induced Urticaria in Children With Kawasaki Disease

**DOI:** 10.3389/fped.2019.00505

**Published:** 2019-12-06

**Authors:** Hui Zhang, Lin Shi, Xiao Hui Li, Ming Ming Zhang, Yao Lin, Yang Liu, Aijie Li

**Affiliations:** Department of Cardiovasology, Children's Hospital Capital Institute of Pediatrics, Beijing, China

**Keywords:** aspirin, urticaria, leukotriene C4 synthase gene, single nucleotide polymorphism, Kawasaki disease

## Abstract

**Objective:** To investigate the distribution of the single nucleotide polymorphism (SNP) of LTC4S A-444C in children with Kawasaki disease in northern China and determine whether LTC4S A-444C SNP is associated with aspirin-induced urticaria (AIU).

**Methods:** The clinical data of children with Kawasaki disease hospitalized in our center from April 2015 to November 2017 were collected, and fluorescence *in situ* hybridization was used to detect the LTC4S A-444C. According to the genotype, the subjects were divided into three groups: AA genotypes, AC genotypes, and CC genotypes. The incidence of AIU in the three groups was calculated and the relationship between LTC4S A-444C SNP and AIU was analyzed.

**Results:** (1) A total of 574 children with Kawasaki disease were enrolled in the study. The allele frequencies for A, C were 980 (85.4%), 168 (14.6%). (2) Twenty-five cases of AIU in AA genotypes, with a positive rate of 6%, 11 cases of AIU in AC genotypes, with a positive rate of 7.5%, 2 cases of AIU in CC genotypes, with a positive rate of 18.2%. CC genotypes had higher incidence of AIU than that of AA and AC genotypes. However, there was no significant difference among the three groups (*P* > 0.05).

**Conclusion:** The proportion of CC genotypes of LTC4S A-444C in children with Kawasaki disease in northern China is lower than that of AA genotypes and AC genotypes, and the incidence of AIU of CC genotypes is higher than that of AC genotypes and AA genotypes.

## Introduction

Kawasaki disease is a febrile disease predominantly occurring in children under 5 years of age, and coronary artery complications are the most common cause of acquired heart disease in children. So far, the guidelines for the treatment of Kawasaki disease in the United States, Japan and China all indicate that high-dose aspirin should be taken in the acute stage and low-dose aspirin should be taken in the convalescent stage. Aspirin has become the routine and preferred approach in the treatment of children with Kawasaki disease (1–3).

However, clinically, aspirin-induced urticaria (AIU) can occur in children with Kawasaki disease during aspirin administration. In severe cases, drug hypersensitivity syndrome can be induced, which endangers the life of the patient and affects the clinical application. How to predict and prevent AIU early? Previous relevant literature reported the diagnosis of aspirin allergy mainly depended on the collection and evaluation of medical history (4), nevertheless according to SimonRA and the European Research Center, the sensitivity and specificity of diagnosis of aspirin allergy by relying solely on medical history were low (5, 6). Provocation testing is the gold standard for the diagnosis of allergies to aspirin and other non-steroidal anti-inflammatory drugs (NSAIDs). In other words, an allergic reaction is induced by increasing dosage gradually from a small dosage, including oral administration, inhalation, intranasal administration and intravenous administration (7, 8), which was limited clinical application due to severe allergic reactions. *In vitro* tests can avoid discomfort and the risk of severe allergic reactions caused by provocation tests. However, it was not widely used on account of the lack of uniformity of test methods. In recent years, a large-scale study on single-nucleotide polymorphisms (SNPs) initiated by The SNP Consortium (TSC) and the National Human Genome Research Institute has demonstrated that SNPs can provide a new method for the study of the complex polygenic diseases and risk of diseases and drug reactions among individuals. Compared to other genetic makers, SNPs have lots of advantages. Firstly, SNPs are numerous and widely distributed. Secondly, SNPs are stable and easy to be conducted automatic analysis, which is suitable for rapid and large-scale screening (9). Aspirin allergy has been reported to be associated with SNPs in specific genes (10, 11), and one of the most studied gene is Leukotriene C4 synthase gene (LTC4S). Yang Lin et al. (12) reported that LTC4S is associated with aspirin drug reaction that has different distribution among populations. Leukotriene C4 synthetase, the key enzyme to synthesize cysteine leukotriene (Cys-LT), plays a crucial role in the pathogenesis of aspirin allergic asthma (AIA), and its activity is genetically regulated by LTC4S, in the study by Sanak et al. (13, 14). The A−444C polymorphism in the promoter region of LTC4S is positively associated with AIA genotypes in Polish. People with C allele have higher risk of AIA. The role of Cys-LT in the pathogenesis of AIU is similar to that of AIA, but the relationship between LTC4S A-444C and AIU is unclear.

It is important for clinician to obtain the drug-sensitive variation information of patients before clinical medication. Compared with adults, pediatric clinical practice is more urgent for the early warning of adverse drug reactions and individual differentiated medication needs. So far there are many studies on the gene diagnosis of aspirin allergy in adults. Among the Han Chinese in Beijing (CHB), the majority of LTC4S A-444C are AA genotypes, followed by AC genotypes, and CC genotypes are the least. The polymorphism and distribution of this gene in children with Kawasaki disease has not been reported. In this study, LTC4S A-444C was detected in children with Kawasaki disease who took aspirin in our hospital, and the correlation between LTC4S A-444C SNP and AIU was analyzed.

## The Research Objective

Children with Kawasaki disease were admitted to our hospital, from April 2015 to November 2017.

### Inclusion Criteria

According to the diagnostic criteria mentioned in the Guidelines for Diagnosis and Treatment of Kawasaki Disease (1, 2):➀ Fever for at least 5 days and four or more of the five major clinical features (i.e., bilateral conjunctivitis without effusion, cervical lymphadenopathy, oral mucosal changes, polymorphous rash, and swelling or redness of the extremities); ➁ Fever for ≥5 days and <4 principal features can be diagnosed as Kawasaki disease when coronary artery complication is found by echocardiography or coronary angiography;➂ Fever for ≥5 days and accord with two or three principal clinical features, or infant has fever for ≥7 days but no other reason can explain, combining of laboratory indicators that can be diagnosed as incomplete Kawasaki disease.

### Exclusion Criteria

➀ Patients who had a history of aspirin allergy themselves or their families. ➁ Patients who were not suitable for aspirin due to co-infection with influenza or varicella. ➂ Patients developed rashes during treatment, except for aspirin allergies, including intravenous immunoglobulin resistance, virus infection, and hemophagocytic syndrome etc. ➃ Patients who refused to participate in the study.

### Treatment

After the diagnosis of Kawasaki disease was confirmed (course of 6–10 days), the patients were received intravenous immunoglobulin (IVIG) 2 g/kg.d and oral high-dose aspirin (30–50 mg/kg.d, Tid). Patients recovered normal temperature for 3 days and inflammatory indicators returned to normal, low-dose aspirin (3–5 mg/kg.d, Qd) was given orally.

### The Diagnostic Criteria of AIU

AIU was considered when the occurrence of new rash after the subsidence of the original rash or aggravation of rash during aspirin administration, then AIU was confirmed by the consultation of fixed observers with a dermatologist, also when rash subsides after discontinuation of aspirin and effectiveness of anti-allergic treatment with cetirizine or chlorpheniramine.

The differential diagnosis of AIU in the study includes IVIG resistance, viral infection, and hemophagocytic syndrome. IVIG resistance presents with not only rash but also fever, and other Kawasaki disease clinical manifestation, moreover, withdrawal of aspirin and anti-allergic treatment did not work to rash. Viral infection is another cause resulted in rash in this situation, but which usually presents with rash and other clinical symptoms, such as fever, cough, or diarrhea. Hemophagocytic syndrome can occur in patients with severe Kawasaki disease, which presents with not only rash, or fever, but also abnormal laboratory tests.

## The Research Method

### Detection of the LTC4S A-444C Genotype

Two milliliters of venous blood of all research subjects were collected in the morning fasting and placed in an EDTA anticoagulant tube. LTC4S A-444C genotype was performed by real-time fluorescence quantitative PCR detection system. The genotype was analyzed by the fluorescence detector (product model: TL998A, production enterprise: Xi'an Tianlong Technology Co., Ltd.), in which a fluorescent Taqman probe was designed for 113LTC4S (A > C) of SNP site. Gene detection results include three genotypes of AA, AC, and CC.

### Clinical Data

Rash and other clinical manifestations during aspirin administration were collected.

### Statistical Methods

SPSS 22 software was used for statistical analysis. χ^2^-test was used to compare the distribution characteristics of alleles A and C. The Fisher's exact test was used to compare the poly-sample rates. Statistical significance was set at *P* < 0.05.

## The Research Findings

### General Information

During the period of the study, total 651 children with Kawasaki disease hospitalized in our department, including 586 cases of complete Kawasaki disease and 65 cases of incomplete Kawasaki disease, in which 37 cases with a history of aspirin allergy themselves or their families, 13 cases who were not suitable for aspirin due to influenza or varicella, 10 cases of IVIG resistance, 6 cases of virus infection and 11 cases who refused to participate in the study, thus 574 cases were included in the study. Three of them recovered normal temperature after admission and didn't receive IVIG or high dose ASP, only with low dose aspirin. Sixty patients diagnosed as IVIG-resistance, which required retreatment with IVIG 2 g/kg (32 cases), steroids (25 cases) or infliximab (3 cases). The age ranged from 0.25 years old to 9.58 years old, with an average age of 2.1 years old. Age distribution of each group: AA group ranged from 0.24 years old to 9.58 years old, with an average age of 2.42 years old; AC group ranged from 0.33 years old to 7.5 years old, with an average age of 2.25 years old; CC group ranged from 0.5 years old to 3 years old, with an average age of 1.58 years old. There were 348 boys and 226 girls. The ratio of males to females was 1.5:1.

### Characteristics of Gene Distribution

The frequencies of A and C alleles in children with Kawasaki disease were as follows: The frequencies of the A allele were 980 (85.4%). The frequencies of the C allele were 168 (14.6%). The frequency of the A allele was higher than that of the C allele. Compared with different populations in PharmGKB database (15), it could be found that East Asians χ^2^ = 0.057, (*P* > 0.05), South Asians χ^2^ = 3.2, (*P* < 0.01), Europeans χ^2^ = 53, (*P* < 0.01), and Africans χ^2^ = 66 (*P* < 0.01). We concluded that the distribution of the A and C alleles in children with Kawasaki disease in northern China was not different from that in Europe and Africa. The frequency of C allele was lower than that of Europeans and higher than that of Africans, which may be related to the genetic backgrounds of different races (Table 1).

**Table 1:** Distribution frequency of LTC4S alleles in children with Kawasaki disease in northern China and other races (15).

	**Allele**		**Total**
**Population**	**A (*n*, %)**	**C (*n*, %)**	***n* (100%)**
Northen China	490 (85.4%)	84 (14.6%)	574 (100%)
African_1_	1,268 (95.9%)	54 (4.1%)	1,322 (100%)
European_2_	692 (68.8%)	314 (31.2%)	1,006 (100%)
East Asian_3_	856 (84.9%)	152 (15.1%)	1,008 (100%)
South Asian_4_	800 (81.8%)	178 (18.2%)	978 (100%)

*Comparison with Africans: $\chi_{\text{1}}^{\text{2}}$ = 66, (P_1_ < 0.01), υ = 1; Comparison with Europeans: $\chi_{\text{2}}^{\text{2}}$ = 53, (P_2_ < 0.01), υ = 1; Comparison with East Asians: $\chi_{\text{3}}^{\text{2}}$ = 0.057, (P_3_ > 0.05), υ = 1; Comparison with South Asians: $\chi_{\text{4}}^{\text{2}}$ = 3.2, (P_4_ > 0.05), υ = 1*.

### Aspirin-Induced Urticaria and Genotype

In this study, there were 417 (73%) AA genotypes, 146 (25%) AC genotypes and 11(2%) CC genotypes. Compared with the Beijing Han Population in Genotype Database, there was no significant difference in the distribution of genotypes between the two groups of population (Table 2). AIU arose from 25 cases of AA genotypes, 11 cases of AC genotypes and 2 cases of CC genotypes, and the positive rate of each genotype was 6, 7.5, and 18.2%, respectively. The positive rate of AIU in AA genotypes was lower than those in AC genotypes and CC genotypes. There was no significant difference among the AA, AC, and CC genotypes (Table 3). Three groups of samples were tested by PASS.15.0.5 software, and it showed the statistic power was low (<80%). Therefore, it did not reach the conclusion whether LTC4S A-444C SNP is associated with AIU in children with Kawasaki disease in northern China in this study.

**Table 2:** Han Chinese in Beijing and children with Kawasaki disease.

	**Genotype (*****n*****, %)**			
	**AA**	**AC**	**CC**	**Total**
Han Chinese	71 (68.9%)	30 (29.1%)	2 (1.9%)	103 (100.0%)
Kawasaki disease	417 (72.6%)	146 (25.4%)	11 (1.9%)	574 (100.0%)
Total	488 (72.1%)	176 (26.0%)	13 (1.9%)	677 (100.0%)

*χ^2^ = 0.625, (P = 0.732, >0.05), υ = 2*.

**Table 3:** Responses of different LTC4S genotypes to aspirin.

	**The response to aspirin**		
**Genotypes**	**Positive**	**Negative**	**Total**
AA	25	392	417
AC	11	135	146
CC	2	9	11
Total	38	536	574

*Fisher's exact test χ^2^ = 3.132, P > 0.05, υ = 2*.

## Discussion

The present study provided the data of the distribution of LTC4S A-444C in children with Kawasaki disease in northern China. The frequency of C allele was lower than that of A allele, which was consistent with that of East and South Asian. The frequency of C allele in northern China was significantly higher than in Africa and significantly lower than in Europe. There was no significant difference in genotype distribution of AA, AC, and CC between the subjects and Beijing Han population, according with the genetic distribution of Beijing Han population ^1^. In this study, we found that the positive rate of AIU in AA was lower than that in AC and CC, but there was no significant difference among AA, AC, and CC.

Provocation testing is the gold standard for the diagnosis of allergies, but with limited clinical application. LTC4S A-444C may be useful as a marker of the AIU phenotype. Although the present study did not find the significant association between LTC4S A-444C genotypes and AIU, it demonstrated that the CC genotypes frequency of LTC4S A-444C in children with Kawasaki disease in northern China is lower than AA and AC; however the incidence of AIU of CC genotypes is the higher than AA and AC, which suggested that LTC4S A-−444C SNP might relate to AIU in children with Kawasaki disease in northern China. The correlation between LTC4S A−444C SNP and AIU needs to be further studied in the future.

Aspirin-induced urticaria and/or vascular edema are one of manifestations of systemic allergic reactions. The pathogenesis of AIU is still unclear by far. Jingang et al. found that (16), intradermal injection of CysLT can cause skin flushes or rubella, vasodilation, and increasing of vascular permeability. The level of CysLTs in AIU patients was higher than that in aspirin tolerant individuals. Wedi et al. reported that CysLT was released by basophils in AIU patients, but not in aspirin-tolerant patients (17). Kim et al. found that single antihistamine was not effective in the treatment of AIU, while leukotriene receptor antagonists had significant effect on AIU (18). Studies above all showed that leukotrienes played an important role in the pathogenesis of AIU.

Leukotriene C4 synthase is the key enzyme for CysLT synthesis, the activity of which is regulated by LTC4S A-444C. (19). Sanak et al. reported the LTC4S A-444C SNP in the Polish population in 1997. Subsequently, the SNPs were confirmed in both Japanese and American patients (20). With the progress of Gene Detection Technology, there are more and more studies on LTC4S A-444C (21, 22). It was found that the high expression of LTC4S mRNA in peripheral blood eosinophils of patients with C allele increased production of Leukotriene C4 synthase, enhancing the ability of cells to synthesize CysLTs (12). Therefore, it can be inferred that patients with the C variant allele may be more likely to develop AIU. In the Polish population, the C allele accounts for a high proportion of chronic idiopathic urticaria patients and aspirin allergic asthma patients who are positive for aspirin stimulation test (23). In the American and Japanese population, the C allele causes aspirin allergic patients to respond better to leukotriene receptor antagonists, so this allele becomes a high risk factor for aspirin allergy (urticaria, asthma). MSnchez-Borges et al. studied the Venezuelan population (South America) and found that AIU was significantly correlated with the LTC4S A-444C. The AA was more frequent in the healthy control group, while AC and CC were more frequent in AIU patients, and it concluded that the C allele of the A-444C polymorphism is a risk factor for AIU in Venezuelan population. (24). Conversely, Kim et al. and Choi et al. found that there was no significant relationship between the LTC4S A-444C polymorphism and LTC4S activity. The genetic polymorphism of LTC4S A-444C is strongly associated with the AIA phenotype in Polish population, but no significant associations were found in American, Japanese, Australian, Spanish or Korean populations (25, 26).

The limitations of this study: (1) The total sample size is not small, but CC group and allergy group is small, which results in low statistic power and may lead to the deviation of results; (2) Some subjects were excluded from the study because of the history of aspirin allergy or family history of aspirin allergy, reducing the number of potential allergies, which may lead to a deviation of results; (3) AIU is a complex condition which may involve multiple genetic variations and gene-environment interactions, thus analysis of the combined effects of multiple genes and multi-locus could capture more information than analysis of a single susceptibility gene or locus. (4) Aspirin allergy might be time-dependent or dose-dependent, which may affect the results of this experiment.

One of the problems in this study is regarding the diagnosis of AIU. AIU is diagnosed clinically by characteristics of rash, the use of aspirin medical history and effective anti-allergic therapy. This is a single-center study with consistent diagnostic criteria that determined by the consultation of fixed observers with a dermatologist. When the occurrence of new rash again after the subsidence of the original rash or aggravation of rash with exclusion of IVIG resistance, viral infection or hemophagocytic syndrome, AIU is suspected, nevertheless, AIU diagnosis is confirmed when rash subsides after discontinuation of aspirin and effectiveness of anti-allergic treatment. This is the strong point of this study because the concern of diagnostic inaccuracy which sometimes becomes problematic in multi-center studies could have been minimized.

## Data Availability Statement

The raw data supporting the conclusions of this manuscript will be made available by the authors, without undue reservation, to any qualified researcher.

## Ethics Statement

This study was approved by the Ethics Committee of the Capital Institute of Pediatrics (No. SHERLL2018020). Informed consent was obtained from a parent or guardian of each patient before the study.

## Author Contributions

LS and XL contributed conception and design of the study. MZ, YLin, and YLiu organized the database. HZ performed the statistical analysis and drafted the manuscript. AL contributed to manuscript revision and approved the submitted version. All authors contributed to manuscript revision and approved the submitted version.

### Conflict of Interest

The authors declare that the research was conducted in the absence of any commercial or financial relationships that could be construed as a potential conflict of interest.

## Footnotes

^1^https://www.ncbi.nlm.nih.gov/variation/tools/1000genomes
